# Poster Session I - A139 EXPLORING THE ROLE OF COMMUNITY SUPPORTS IN IMPROVING OUTCOMES FOR RECENTLY DISCHARGED PATIENTS WITH INFLAMMATORY BOWEL DISEASE: A SYSTEMATIC REVIEW

**DOI:** 10.1093/jcag/gwaf042.139

**Published:** 2026-02-13

**Authors:** M C MacDonald, N Willett, J Jones

**Affiliations:** Internal Medicine, Dalhousie University, Halifax, NS, Canada; Internal Medicine, Dalhousie University, Halifax, NS, Canada; Internal Medicine, Dalhousie University, Halifax, NS, Canada

## Abstract

**Background:**

Inflammatory Bowel Disease (IBD), including Crohn’s disease (CD) and ulcerative colitis (UC), is a chronic relapsing condition with significant morbidity. Canada has one of the highest global prevalence rates, projected to reach 1.1% by 2035. The post-discharge period is a vulnerable transition for IBD patients, who face challenges with medication, diet, and psychological distress (IBD-PD). Poorly coordinated care can lead to readmissions and mental health decline. Community-based supports such as structured follow-up, telehealth, and access to nutrition or mental health services may improve outcomes, but their effectiveness has not been systematically reviewed.

**Aims:**

To evaluate the effectiveness of community-based post-discharge interventions for adults with IBD in reducing readmissions, improving disease control, and enhancing quality of life and psychological well-being.

**Methods:**

This review followed PRISMA guidelines. We included studies of adults (≥18 years) discharged after an IBD-related hospitalization who received community-based post-discharge supports, including structured follow-up, telehealth, dietitian, mental health, or peer support. Usual care without structured follow-up served as the comparator. Outcomes were readmission, disease control, quality of life, and IBD-PD. Eligible designs were randomized, cohort, and qualitative studies in English. Five databases (MEDLINE, Cochrane, Scopus, Embase, CINAHL) were searched. Two reviewers independently screened, extracted data, and assessed bias using Covidence and ROB-2. Certainty of evidence was rated with GRADE, and findings summarized narratively due to heterogeneity.

**Results:**

Thirteen studies met inclusion criteria, including randomized, cohort, and mixed-method designs. Interventions involved structured outpatient programs, telehealth follow-up, and multidisciplinary care with dietitian or psychological support. Follow-up ranged from 30 days to 12 months. Compared with usual care, structured post-discharge programs were linked to lower readmission rates and improved disease control. Most also showed better quality of life and satisfaction, particularly with telemonitoring and multidisciplinary models. Qualitative data highlighted improved confidence, continuity of care, and reduced anxiety. Risk of bias was moderate, mainly due to small samples and lack of blinding. Certainty of evidence ranged from low to moderate.

**Conclusions:**

Community-based post-discharge interventions for IBD may reduce readmissions and improve patient-reported outcomes. Structured follow-up, especially telehealth and multidisciplinary care, strengthens continuity of care during this transition. Evidence remains limited by heterogeneity and moderate bias, underscoring the need for larger, high-quality trials with standardized outcomes.

**Funding Agencies:**

None

